# Proving of the X Ray*Summary or second part of a paper read before The International Hahnemannian Association June 30th, 1897.

**Published:** 1897-08

**Authors:** B. Fincke

**Affiliations:** Brooklyn, N. Y.


### PROVING OF THE X RAY.*

B. Fincke, M. D., Brooklyn, N. Y.

### MIND AND DISPOSITION.

Mental irritability.

Clearing up of mental function after sharp, stabbing pain in left temple staggering him, the heart feeling the impulse immediately.

Mental depression after snatches of sleep, for twelve days.

Mental processes not clear, writes wrong words in letters.

Mental condition upset during profuse menstruation; would like to kill somebody.

Misanthropy during renal colic; did not want to answer questions, did not want to see anybody or talk with anybody, being completely prostrated.

### HEAD.

A feeling at right external orbital margin as if it might be an ache, immediately (the first symptom observed).

A pressing across forehead with a miserable feeling generally as though going to be sick.

Awoke with headache next morning, continuing at intervals through whole day.

Sleepy with headache.

Headache gradually extending to frontal region, worse in centre of forehead.

Sense of pressure in centre of forehead.

Dull headache in morning, worse when stooping and after rising.

Headache and soreness worse toward afternoon.

Head feels empty as though scraped, worse at night in bed.

---

* Summary or second part of a paper read before The International Hahnemannian Association June 30th, 1897.

Sensation of fullness of head with slight full feeling in ears, worse in right ear.

Pain in right side of head above temple.

Threatening headache, pressure through temples.

Headache across brow and a different pain in temples.

Pain in right side of head, passing around base of brain to right ear, followed by paralytic numbness.

Pain in left temple, worse at 8 P. M.

Sharp stabbing in left temple, followed by clearing up mental function, the heart immediately feeling the impulse.

Feeling in head and nose as if coryza would set in with slight desire to sneeze.

Sense of pressive fullness, starting from posterior prominence of vertex in a central straight line to bridge of nose, followed by fullness in entire vertex extending to bridge of nose.

Aggravation of fullness over vertex worse along the centre to nose and when stooping over.

Disagreeable fullness of head all morning, with bland coryza and stoppage of nostrils.

Aching on top of head across along coronal suture on blowing nose and after it.

Constant ache in vertex, worse on awaking, also on coughing, sneezing, or head low.

Drawing in and across brow approaching nose and resting there, immediately.

Pain across brow over bridge of nose.

Heavy pressure on vertex, as from a hand (old symptom, absent a year).

Sticking pains in different parts of head and face.

Morning, awaking earlier than usual, with dull aching in left occiput, and immediately in left sacro-iliac region, lasting fifteen minutes.

Suddenly sharp aching in left occiput on small area, recurring two or three times at intervals of some hours, without regularity.

Violent sick headache second day of menses, pulsating pressure outward in forehead as if bursting, vomiting, pain relieved by hot water applications.

### EYES.

Bearing down of eyelids.

Eyeballs sore.

Sensation in right eye as if bulging.

Right eye sore to touch.

Congestion of eyes in forenoon, worse on rising.

On closing eyes in dark sees old men's and women's ugly faces.

Eyelids heavy and sleepy.

### EARS.

Sore pain in cartilage of left ear when pressing it, later in right ear.

Pressing outward in ears.

Buzzing in right ear, with pressure extending to temples.

Fullness in ears, worse in right ear, worse by inserting finger.

Fullness in ears, more in right, and fullness in head.

Intermittent noises as of deep steam-whistle in left ear, and singing in head.

Ears more clear from singing and dullness of hearing than for many years, an improvement lasting up to this day. Healing action.

### NOSE.

Bloody mucus from nose.

Sensation of sulphur-vapor in nose, with much sneezing.

Sulphur-vapor sensation in throat and nose.

Sensation in head and nose as before coryza.

Nose stopped on left side.

On blowing nose and after it aching across top of head along coronal suture.

Sensation of hyperæsthesia or nervous concentration in bridge of nose.

Nasal discharge thin.

Flowing coryza with stoppage of nostrils and fullness in head, forenoon.

### FACE.

Dull pain in right upper jaw.

Paralytic feeling in right cheek, preceded by chills going down the back.

Slight electric current sensation in left side of tongue and face, passing over and disappearing in right side of face.

Red, smooth eruption on right side of face.

Stinging pains in different parts of head and face.

### MOUTH.

Tongue dry, rough, sore, and scraped.

Scraping pain in lateral incisor, aggravated by noises and jarring of cars.

Teeth covered with a gray-green deposit.

Tongue slightly coated.

### THROAT.

Sulphur-vapor sensation in nose and throat.

Throat painful on swallowing.

Sense of painful lump about left tonsil, worse when swallowing.

Indescribable sensation in œsophagus immediately.

Foul breath.

After eating at noon, sensation of a long and narrow foreign body lodged in pharynx, removed by swallowing, aggravated by swallowing. After this passed away, pharynx felt hollow and sore on deglutition.

### APPETITE.

No appetite for anything except sweet pudding or cake.

Aversion to meat.

Ate nothing for three days.

No appetite for breakfast (old symptom, absent for years).

Appetite diminished.

Desire for sweets.

Most distress after midday and evening meal.

No hunger, goes until he feels faint.

Can eat plenty, but does not enjoy it.

More thirst than usual.

Thirst for cold drinks, though nothing tastes good.

Bad taste in forenoon.

Bitter taste.

### NAUSEA, VOMITING.

Nausea and vomiting, with profuse sweat after immense stool at 4 A. M., seven days after taking.

Slightly sick at stomach.

Vomiting ceased, though colic pain remained in lower abdomen for twelve days.

### ABDOMEN.

Abdomen distended with full feeling (Pulsatilla, which a year ago helped for months a train of symptoms, had only very slight effect).

Flatulence with ineffectual desire for stool.

Heaviness and fullness in lower abdomen after eating small quantity.

Uneasy feeling in lower abdomen at intervals, like fermentation, as though diarrhœa would follow.

Wind around heart, flatus and flatulence as though diarrhœa would commence.

Drawing gnawing in region of appendix, as though inflammation were beginning.

Sensation in right lower abdomen as though bubbles were forming and wanting to burst. (Relieved by Taraxacum for twenty-four hours.)

Flatulence with ineffectual desire for stool.

Colicky pains in right lower abdomen, sometimes extending behind the hip, and retained urine.

Moving aggravates the colic pains, especially on right abdomen, which seemed adherent to walls, feeling as if torn off. Had to keep a large towel-pad between abdomen and thigh to support abdomen and prevent excruciating pain.

Pain in right side just above the crest of ilium, with an irritation in larynx causing cough in morning after rising and before eating; eating relieved cough, and the side felt bruised when bending or jarring or pressing it.

STOOL.

Catarrhal inflammation of rectum with discharge of mucus slightly bloody after action of bowels in course of two or three days (a trouble he had once before).

Seven days after taking medicine, roused out of bed at 4 A. M. with desire for stool and complete evacuation of immense quantity, followed by nausea and vomiting, profuse sweat, pain in right lower abdomen, retained urine, vesical tenesmus continuing for twelve hours, until 4 P. M., after which urine became freer, vomiting ceased, colic pains remained for twelve days.

Stools green, though normal in consistency.

On straining to stool a sore feeling in nates.

Ineffectual urging to stool with flatulence.

URINE.

Retained urine, vesical tenesmus after enormous evacuation and vomiting, for twelve hours to 4 P. M.

Seven days from beginning of attack return of renal colic with vomiting, continuous nausea and retention of urine on left side. A clutching pain from left kidney to bladder that never relaxed for four hours, causing the wildest demonstration of agony, ghastly face, blue rings around sunken eyes, cold sweat with trembling, frightful restlessness, urine retained in ureter (giving a perfect picture of Tabacum, which relieved at once). As soon as the pain in ureter relaxed after it, urine was filling up the bladder and then passed without pain. Some spasm remained, and was relieved by Cantharis in five minutes. (She had something of a like trouble a year ago, but not so severe as this, only a strangury.)

Did not want to answer questions, did not want to see anybody or talk to anybody, complete misanthropy. Great prostration followed this attack.

Frequent urination, worse after getting into bed.

Pressure as from congestion about kidneys.

### SEXUAL ORGANS.

Sexual desire lost in man.

Testes relaxed, impotent feeling.

Unnatural or disgusting, lewd dreams on several nights, or several times in one night.

Pain darting upward in region of left ovary, when sitting, walking, or standing.

Menses dark green one day.

Second day of menses a violent sick headache, pulsating pressure outward in forehead as if bursting, vomiting (pain relieved by hot water application).

After cessation of menstruation for six months it reappeared again, violent, profuse, without pain, but prostrating, the flow lasting several days.

Three or four days before menstruation felt from waist down as if bursting, with distention and weight in abdomen and wanted to hold herself up, relieved by the flow. Mental condition upset, she would like to kill somebody. (Three years ago she proved Uranium-nitrate. Up to that time she had never missed a regular menstruation, but irregular ever since.)

### AIR PASSAGES.

Cough caused by palpitation during evening.

In morning and after rising and before eating a cough, caused by irritation in the larynx, accompanied by a pain in right side just above the crest of ilium; eating relieved cough, but side felt bruised when bending, jarring or pressing it.

In less than a week a cough with tearing sensation in bronchi, hoarseness, palpitation.

Phlegm of greenish hue causing cough in crawling up.

On rising in morning expectoration of considerable white mucus.

Much expectoration of tough mucus as large as finger's ends, whitish like gelatine, raised easily.

Raising of jelly-like mucus, worse in forenoon, tenacious and tough.

Grayish expectoration.

### CHEST.

Wandering sticking pains in chest, worse on right side.

Stitching pain in right upper chest, going through to upper part of scapula.

Sense of contraction across middle chest.

Contraction of chest at night, relieved by belching.

Left chest bulged out like enlargement of heart. (Old symptom of inflammatory rheumatism twenty-five years ago.)

Sensation of drops of water trickling down on inside of chest.

Sensation in left side as if fingers pressed costal cartilages in, followed by sensation as if something broke inside, with temporary relief.

### HEART.

Palpitation during evening, causing cough.

Pulsations greatly diffused and violent, provoking an exclamation which was suppressed, accompanied with a desire to walk in open air, and entirely relieved by lying down.

Sharp pain at apex of heart, better by lying on left side.

Palpitation of heart.

Palpitation with a cough with tearing sensation in bronchi and hoarseness.

Painful bulging of left chest over cardiac region (old symptom of inflammatory rheumatism twenty-five years ago).

Heart's sounds keep him awake while lying on the left side.

Dull and constant soreness around heart and worse in legs and arms.

Wind around heart, flatus and flatulence as though diarrhœa would begin.

### BACK.

Waking earlier than usual by dull aching in left occiput and immediately in left sacro-iliac region, then posterior thigh and calf for fifteen minutes.

Pain in dorsal region.

Lame and stiff in the back.

Aching whole length of spine.

After waking at daybreak next morning, sensation in dorsal region of spine, as if its convexity were interior and drawn far forward, lasting but a minute, and followed by a very uncomfortable feeling in that region, lasting a much longer time.

Sore feeling across loins as after a heavy cold.

Sensation as if a cool drop of sweat were going down left side of spine.

Soreness in small of back.

A paralytic sensation extending from spine down left leg.

Rheumatic-like fever in trunk, steady dull pain going steadily from trunk to legs and finally to heels, worse in left knee, worse stepping on heel, or underside of heel (old symptom of inflammatory rheumatism twenty-five years ago).

Pressure in small of back, as from congestion about kidneys.

### UPPER EXTREMITIES.

Magnetic thrill in right hand extending up forearm.

Tingling in both arms as of electric current, or as if asleep.

Intermittent, slightly burning, rheumatic-like pain in right carpo-metacarpal articulation, extending from index finger up outside of right forearm.

Rheumatic pain in left wrist and forearm.

Rheumatic twinge in last two phalangeal articulations of index and middle fingers for a short time in forenoon.

Aching extensor muscles of right forearm going up to shoulder.

Rheumatic pain in right wrist and arm.

Tingling like pins and needles in left hand.

Tingling in right hand.

Can't hold things in left hand, powerless or clumsy.

Palms of hands which were rough and scaly and bleeding at times became smooth and natural during the proving. Healing action. (Afterward they went back to former state when general health improved.)

### LOWER EXTREMITIES.

Lower part of both legs asleep, tingling, as of electric battery, more in right, immediately.

Sciatic pain in right hip.

Rheumatic pains in limbs.

Dull aching posterior aspect of thigh and calf in morning, from above downward.

Pain in right sciatic nerve on walking.

Rheumatic pains in front of right thigh.

Drawing aching discomfort in right thigh through hip and knee down through toes, immediately.

Feeling as if somebody were drawing icy hands over thigh downward, aggravatingly slow. (This occurred first twelve years ago after a nervous shock and did not return for five years.)

Varicose veins inside of knees and legs with swelling and soreness (returned after years).

Had always heat and swelling of feet in spring, which is better now although it is spring. Healing action.

On inside of left knee sensation in different spots painful as if hairs were being pulled, worse walking, better rubbing, or scratching.

Rheumatic-like fever in trunk, steady dull pain going steadily down to legs, and finally to heels, worse in left knee, worse stepping on heel, or underside of heel (old symptom of inflammatory rheumatism twenty-five years ago).

### FEVER.

Chills as soon as beginning to sleep, running up back, preventing sleep.

Chilly whilst undressing in warm room.

Chilliness on moving or from draft.

Chill going down back followed by paralytic feeling in right cheek.

Wave-like sensation as if it would break out in perspiration.

Profuse perspiration on getting into bed, keeping him awake.

Spells of feverishness, perspiration, and weakness.

### SLEEP.

Kept awake by heart's sounds while lying on left side.

Sleepy with headache.

Only snatches of sleep during these twelve days, always followed by mental depression, during suffering from nephralgia.

As soon as she begins to sleep chills running up back preventing sleep.

Sleepy, but unable to sleep for some hours after retiring.

Sleeplessness constant and troublesome (relieved permanently by a bottle of Pabst Malt Extract).

Drowsy all night while sitting up.

Drowsiness leaves the instant when lying down, so cannot sleep.

Goes to sleep on right side, but wakes on left side.

Symptoms worse when getting into bed, worse after sunset.

Profuse perspiration on getting into bed, keeping him awake.

Sleeping during day.

Waking frequently at night from no apparent cause.

All symptoms worse in bed.

Frequent urination, worse after getting into bed.

(Symptoms better after Bell. or Acon., for a short time, better from ale, which puts him asleep.)

Dreams of distressing nature.

Sleep not unrefreshing, though apparently dreaming all night.

Awaking with headache lasting all day.

Recurrence of a dream which used to trouble her many years ago. (This dream was repeated at wide intervals over twenty years ago.) Now it came every night for five times.

Dreams of strife, busy dreams.

Very vivid lewd dreams, repeated night after night.

Unnatural lewd and disgusting dreams occurring on several nights and several times in one night.

### SKIN.

Reappearance of an old slightly-pimply eruption on left side of forehead.

Return of a slight eruption on outside of lower legs, burning when scratched, worse after scratching.

### GENERAL SYMPTOMS.

General tired and sick feeling.

Persistent exhaustion and languor not attributable to spring.

Lame and sore all over.

Trembling all over.

Body tired and exhausted.

Nervous powerlessness in limbs.

Sensation of swooning at times during day and night, feeling like dying.

Prostrated for a long time.

All symptoms worse in bed.

All symptoms worse toward evening and at night.

Mental and physical aggravation markedly pronounced toward evening.

Symptoms intermittent in character, the first observed symptoms have all returned at irregular intervals.

Muscles feel soft subjectively.

Generally worse in open air.

Twitching internally in various parts.

Symptoms gradually increasing in severity after noon.

### CASE OF DR. JOHN B. CAMPBELL.

A patient for three years has gout, which is the summing up of a history of suppressed chills. He got X ray 30th centesimal, which seems to have helped him somewhat. Swelling of legs and knees disappeared, after being present for weeks. Some of the pains were better. 1897, March 29th, 2.30 P. M., took X ray 30th cent., one powder, at 5 P. M. the second. No symptom. At 8 P. M. the third, which produced little itching. At 10 P. M. took a hot bath with some improvement, and at 11 P. M. the fourth powder, all of the 30th cent.

After that decided glow of heat all over body with some itching.

Little rash on both legs, with decided pricking on both legs from knees to toes.

Pain in region of spleen for one hour, which passed away in bed, during which time no twitching or jerking of muscles, could stretch out both legs comfortably.

Very good night's sleep.

March 30th, 7 A. M. Slight perspiration on legs from knees to toes.

March 31st. Took the remaining powder of same potency.

Some itching on left shoulder-blade and arm from shoulder to elbow with considerable increase of pain in elbows and hands and sensation of heat all over body, but no fever.

Stiffness of back of neck, necessitating moving of whole body to turn his head.

Slight rash on left leg above the knee.

Some itching of arms and chest in bed, especially when beginning to get warm.

Could walk a little better the first two days after taking the medicine.

More thirst than usual.

Had no chill since 21st until to-day, when he had a slight chill from 2 to 4 P. M., no fever, thirst, or sweat.

Appetite and bowels extremely good.

Some itching or prickling pains above knees.

The swelling from legs and feet has gone down immensely, though pain does not abate.

[The individual reports of the provers of the X-ray as originally appearing in Dr. Fincke's paper are quite long and very interesting. It has not been deemed necessary to reproduce them here. Those who wish to consult them are referred to the proceedings of I. H. A. for 1897.—Ed.]

